# The role of titin and extracellular matrix remodelling in heart failure with preserved ejection fraction

**DOI:** 10.1007/s12471-016-0812-z

**Published:** 2016-02-17

**Authors:** C. Franssen, A. González Miqueo

**Affiliations:** ICaR-VU, VU University Medical Center, Van der Boechorststraat 7, 1081 BT Amsterdam, The Netherlands; Center for Applied Medical Research, University of Navarra, Program of Cardiovascular Diseases, Pamplona, Spain

**Keywords:** HFpEF, Extracellular matrix, Titin, Biomarkers, Myocardial stiffness, Myocardial remodelling

## Abstract

Heart failure with preserved ejection fraction (HFpEF) is characterised by a high incidence of metabolic comorbidities that share the potential to induce both systemic and coronary microvascular inflammation and oxidative stress. These pathophysiological alterations contribute to increased passive stiffness of the myocardium and to diastolic dysfunction, both hallmarks of HFpEF. Passive myocardial stiffness depends mainly on two components: the extracellular matrix (ECM) and the cardiomyocytes. Quantitative and qualitative changes in collagen metabolism leading to myocardial fibrosis determine the ECM-based stiffness of the myocardium. Different noninvasive diagnostic tools to assess myocardial fibrosis are being developed, some of which have demonstrated to correlate with clinical status and prognosis. Cardiomyocytes mainly alter the passive stiffness through alterations in the giant myofilament titin, which serves as a spring. By modifying its phosphorylation state or by direct oxidative effects, titin determines cardiomyocyte-based passive stiffness. Probably the relative importance of cardiomyocyte-based changes is more important in the beginning of the disease, whereas ECM-based changes become more prominent in the more advanced stages. The present review focuses on these changes in ECM and cardiomyocytes in HFpEF and their potential prognostic and therapeutic implications.

## Introduction

In contrast to heart failure with a reduced ejection fraction (HFrEF), patients with heart failure with a preserved ejection fraction (HFpEF) still do not benefit from evidence-based treatment options in the absence of a profound knowledge about its pathophysiology. Therefore, HFpEF therapy is aimed at comorbidities and at reducing signs and symptoms of congestion [1]. Indeed, just over a decade ago, knowledge about myocardial structure and function in HFpEF was very poor [2]. In the following years many studies addressed epidemiological, clinical and fundamental aspects in HFpEF. This eventually led to a novel paradigm in HFpEF pathophysiology with a central role for metabolic comorbidities on top with downstream effects such as inflammation and oxidative stress, eventually interfering with normal myocardial function [3]. Indeed, non-cardiac comorbidities such as obesity, arterial hypertension and diabetes mellitus are highly prevalent in HFpEF [4]. These comorbidities generate a chronic, systemic inflammatory state and diverse markers of inflammation have been found to be associated with the diagnosis and prognosis of HFpEF [5, 6]. According to this HFpEF paradigm, the observed systemic inflammation also leads to myocardial microvascular endothelial activation and oxidative stress [3]. At the cardiac level, this endothelial inflammation and oxidative stress induce myocardial stiffening.

Although there are many other pathophysiological findings in HFpEF, as recently discussed comprehensively elsewhere [7], this review will focus on myocardial abnormalities and the interplay between changes in the extracellular matrix (ECM) and the cardiomyocytes (and specifically titin) that cause myocardial stiffening.

## Increased myocardial stiffness in HFpEF

The signs and symptoms of HFpEF are based on increased myocardial stiffness, leading to diastolic left ventricular (LV) dysfunction, which is defined as the inability of the heart to fill to an adequate end-diastolic volume at acceptably low pressures in the absence of endocardial or pericardial disease [8]. Although practically all patients with heart failure, regardless of LV ejection fraction (LVEF), have diastolic dysfunction to a greater or lesser degree, HFpEF patients have a non-dilated left ventricle with globally preserved systolic function (LVEF > 50 %). Diastolic dysfunction is either diagnosed invasively (by measuring elevated pulmonary capillary wedge pressure, LV end-diastolic pressure or prolonged LV isovolumic relaxation) or noninvasively with tissue Doppler echocardiography [9, 10].

**Fig. 1:**
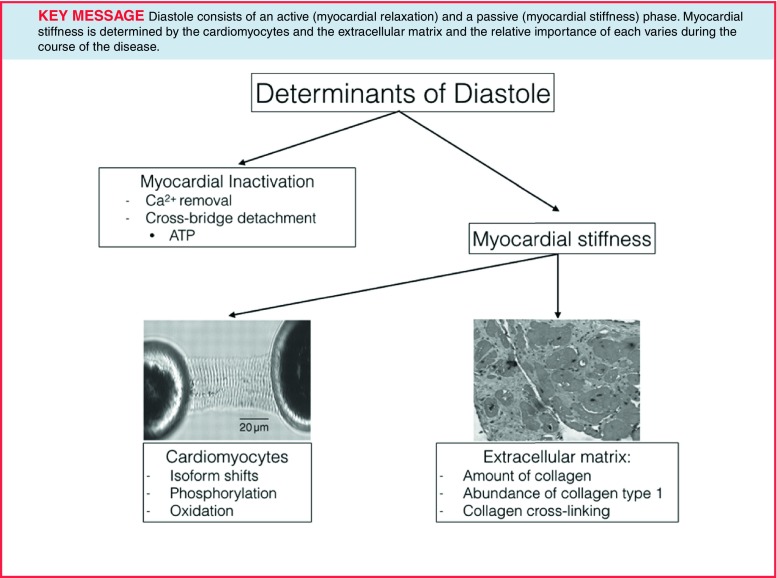
Determinants of diastole. LV diastole can be subdivided into two components: myocardial inactivation and myocardial stiffness. This myocardial inactivation is the consequence of dissociating contractile myofilaments and calcium reuptake into the sarcoplasmic reticulum. Myocardial stiffness can be attributed to the viscoelastic properties of the myocardium [11]. The two myocardial compartments that regulate the viscoelastic properties and hence myocardial stiffness are the ECM and the cardiomyocytes, in which the giant protein titin plays a key regulatory role. (Modified with permission from [59])

LV diastole can be subdivided into two components: myocardial inactivation and myocardial stiffness. This myocardial inactivation is the consequence of dissociating contractile myofilaments and calcium reuptake into the sarcoplasmic reticulum. Myocardial stiffness can be attributed to the viscoelastic properties of the myocardium (Fig. 1; [11]). The two myocardial compartments that regulate the viscoelastic properties and hence myocardial stiffness will be discussed next: the ECM, namely the collagen network, and the cardiomyocytes, in which the giant protein titin plays a key regulatory role.

## The extracellular matrix in HFpEF

### Quantification of collagen content

The first indications about HFpEF pathophysiology were based on human myocardial biopsy samples, which showed myocardial fibrosis with an increased collagen volume fraction (CVF) in HFpEF patients compared with controls [12]. These findings were recently confirmed in patients with an ante-mortem diagnosis of HFpEF expressing more myocardial fibrosis on autopsies than in age-matched controls [13]. Besides, it was demonstrated that an inflammatory trigger, such as is present in HFpEF, can induce the differentiation of myocardial fibroblasts into collagen-producing myofibroblasts after stimulation with transforming growth factor-β [14].

**Fig. 2:**
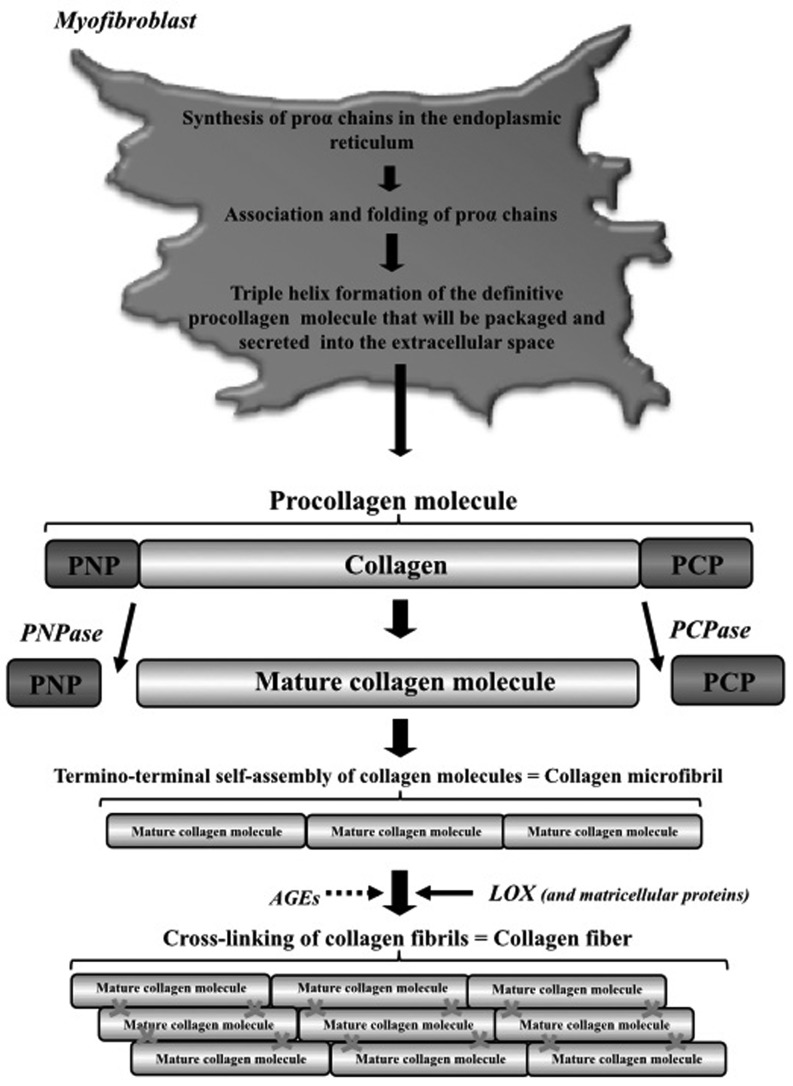
Schematic representation of the process of collagen fibres formation. (Adapted from [60] with permission). *PCP* procollagen carboxy-terminal propeptide, *PNP* procollagen amino-terminal propeptide, *PCPase* PCP prcollagen carboxi-pepdtidase, *PNP* procollagen amino-peptidase, *AGEs* advanced end-glycation products

However, quantification of the total collagen content with CVF seems to have less functional implications than the relative amount of the stiffer collagen type I over the more compliant collagen type III, or the amount of cross-linked collagen by lysyl oxidase (Fig. 2; [14, 15]). For example, human HFpEF myocardial biopsy samples contained increased levels of collagen type I, enhanced collagen cross-linking and lysyl oxidase expression and these findings were associated with parameters of diastolic dysfunction on tissue Doppler echocardiography [16]. Also, it was demonstrated that HFpEF patients with diabetes mellitus have increased deposition of advanced glycation end-products in the ECM, which are able to cross-link collagen and increase myocardial stiffness (Fig. 2; [17]). Moreover, advanced glycation end-products are known to induce myocardial inflammation [18, 19] and oxidative stress [20]. The relevance of myocardial inflammation and oxidative stress will be discussed in more detail later.

Research has focused on noninvasive techniques to measure LV myocardial fibrosis to avoid the (low) risk of complications and sampling error, both inherent to the procurement of an endomyocardial biopsy. Cardiac magnetic resonance (CMR) imaging, for example, allows for quantification of diffuse myocardial fibrosis by measurement of longitudinal relaxation time (T1 mapping). Several T1 mapping methods have been validated with endomyocardial biopsies to assess diffuse myocardial fibrosis. These techniques include post-contrast T1 mapping, calculation of extracellular volume fraction using MOLLI (Modified Look-Locker inversion recovery) sequences, and equilibrium contrast CMR [21]. Indeed, extracellular volume fraction as a marker of diffuse myocardial fibrosis correlated with impaired diastolic function in HFpEF [22]. On the other hand, numerous biomarkers related to collagen metabolism or its turnover, or molecules integrating cardiac stress injury, inflammation and fibrosis have been studied. However, blood levels of a valid biomarker of myocardial fibrosis should directly correlate with quantitative parameters used to define fibrosis in endomyocardial biopsies [23]. Of all possible candidates, only PICP (the carboxy-terminal propeptide of procollagen type I) and PIIINP (the amino-terminal propeptide of procollagen type III) have been shown to be associated with myocardial fibrosis (Fig. 2; [23]). Possibly different biomarkers of collagen and ECM turnover vary during the transition from being at risk for HFpEF development to more advanced stages of the disease, although further studies are needed to validate these biomarkers and their potential role in HFpEF diagnosis, treatment and prognosis.

### Prognostic relevance of myocardial fibrosis

After fibrosis is detected, its clinical or prognostic consequences need to be established. As mentioned above, extracellular volume fraction can be determined with CMR in HFpEF patients as a measure of diffuse myocardial fibrosis [22]. Extracellular volume fraction correlated with LV end-diastolic and systolic volumes, LV mass, LVEF, peak filling rate and peak ejection rate in HFpEF patients [22]. A significant association has been reported between CMR T1 time (validated in LV endomyocardial biopsies) and cardiac outcomes (hospitalisation for heart failure or death from cardiovascular causes) in HFpEF patients [24]. However, further large-scale studies need to establish the prognostic relevance of these findings and their value in clinical decision-making.

The number of biomarkers that are currently being studied or developed reflecting myocardial fibrosis in HFpEF is growing exponentially and a detailed discussion of this topic is beyond the scope of this review. The most important and promising biomarkers were recently discussed elsewhere [25]. Especially ST2 and galectin-3 have the potential to predict prognosis in HFpEF and, next to this, galectin-3 is related to aldosterone signalling and might identify patients for treatment with aldosterone antagonists [26]. However, in a recent substudy of the RELAX trial, galectin-3 correlated with renal dysfunction and, taking this into account, was not independently associated with the severity of HFpEF [27]. Further research is needed to elucidate the role of biomarkers of fibrosis in HFpEF.

### Functional relevance of myocardial fibrosis in diastolic dysfunction

**Fig. 3:**
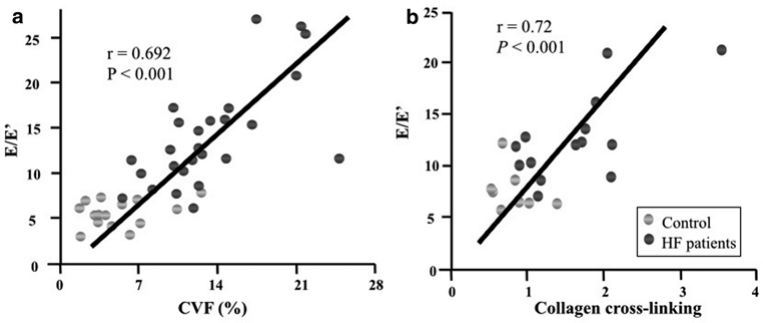
Association of (**a**) collagen volume fraction (CVF) and (**b**) collagen cross-linking with left-sided filling pressures echocardiographically estimated in heart failure patients with preserved ejection fraction (HFpEF). (Adapted from [61] with permission). *E* maximum early transmitral flow velocity in diastole, *E’*, maximum early diastolic velocity of the mitral annulus displacement

Associations of myocardial CVF with parameters related to diastolic dysfunction such as LV end-diastolic pressure [12] or the E:E’ ratio (the ratio of transmitral E velocity to early diastolic mitral annular velocity) [16] have been found in HFpEF patients (Fig. 3). Moreover, associations between collagen-dependent stiffness and pulmonary capillary wedge pressure or left atrial diameter have been reported [28]. However, it has to be considered that LV end-diastolic pressure, LV end-diastolic wall stress and myocardial stiffness modulus, were increased in HFpEF patients versus controls, even in those patients with low CVF, suggesting that myocardial fibrosis is not the sole contributor to LV diastolic dysfunction [12].

In this regard, in HFpEF patients, passive stiffness (F_passive_) of single isolated, membrane-permeabilised cardiomyocytes was shown to be significantly higher during muscle lengthening than in HFrEF, despite increased CVF in HFrEF versus HFpEF [29]. Of course these single cardiomyocyte experiments do not account for ECM-based F_passive_. More recently, functional experiments on small myocardial muscle strips allowed the differentiation between cardiomyocyte- and ECM-based F_passive_. In an HFpEF patient population undergoing coronary artery bypass surgery, force measurements were performed on epicardial biopsy samples obtained during surgery [28]. In these patients, ECM-dependent stress was associated with elevated filling pressures and left atrial dilatation. At higher muscle and sarcomere lengths, increases in ECM-based F_passive_ account for more than two-thirds of total F_passive_ in HFpEF [28], which suggests that collagen serves as a back-up mechanism to prevent supraphysiological stretch [30]. At lower sarcomere lengths, the titin-dependent F_passive_ was also shown to be increased and to correlate with left atrial diameter [28]. The relative contributions of titin and collagen to F_passive_ are therefore dependent on sarcomere length, but the actual operating range of sarcomere length in HFpEF patients is unknown. The giant protein titin forms a unique filament network in cardiomyocytes, which engages in both mechanical and signalling functions of the heart and will be discussed in more detail later [31].

Of interest, the functional relevance of myocardial collagen on diastolic dysfunction may depend on the stage of the disease as illustrated in a ZSF1 (Zucker diabetic fatty/Spontaneously hypertensive heart failure F1 hybrid) rat model in which HFpEF is induced by obesity and diabetes mellitus on top of arterial hypertension [32]. Twenty-week-old HFpEF rats had increased myocardial F_passive_ compared with hypertensive controls (without a HFpEF phenotype). However, CVF was not increased in these animals compared with the control group and the increase in F_passive_ was attributed to a stiffer cardiomyocyte compartment [32].

Finally, as previously mentioned, not only the quantity of collagen but also some qualitative aspects such as the degree of collagen cross-linking [33] or the collagen type I: type III ratio, with collagen type I being stiffer than collagen type III [34], may influence collagen solubility and myocardial stiffness. In this regard, in patients with hypertensive heart failure, collagen cross-linking but not CVF was associated with elevated filling pressures (Fig. 3; [35]). Moreover, an increase in insoluble collagen accounts for the increase in total collagen and ECM-based F_passive_ in HFpEF patients [28]. On the other hand, whereas collagen type I expression was found to be increased in the myocardium of HFpEF patients and associated with the E:E’ ratio, no significant changes were found in collagen type III expression [16].

## Cardiomyocytes in HFpEF

Next to the observed ECM changes, cardiomyocytes also undergo changes in HFpEF. When compared with HFrEF, cardiomyocytes in HFpEF patients are larger and stiffer with higher F_passive_ upon stretch [29]. In cardiomyocytes, the giant protein titin operates as a bidirectional spring and gives stability to the other myofilaments [36]. Titin determines the sarcomeric viscoelasticity, whereas actin and myosin mainly contribute to force generation [37]. Titin is able to modulate cardiomyocyte-based F_passive_ by means of isoform switching, phosphorylation and oxidative modifications [31]. In the adult human heart, titin exists as two isoforms: a longer and more compliant N2BA isoform and a shorter and stiffer N2B isoform. The N2BA:N2B ratio changes during the course of different heart diseases, but in general the ratio increases in eccentric remodelling and decreases in concentric remodelling [38]. However, these changes probably take place gradually during the course of days to weeks and evolve during disease states, whereas phosphorylation and oxidative modifications occur much faster [31].

Titin can be divided into certain regions, and especially the I-band is known to contain two spring elements: the N2-B unique sequence (N2-Bus) and a region rich in proline, glutamate, valine, and lysine (PEVK) [31]. Many serine and threonine residues of titin are already identified as phosphorylation sites for different protein kinases (PK), such as PKA [39], PKC [40], PKG [41], extracellular signal-regulated kinase-2 (ERK2) [42] and Ca2+/calmodulin-dependent protein kinase-II (CaMKII) [43, 44]. Phosphorylation of specific titin sites can alter its distensibility and hence stiffness. For example, in the first studies in human HFpEF biopsy samples, it was observed that *in vitro* administration of PKA decreased F_passive_ in isolated cardiomyocytes, suggesting a titin phosphorylation deficit in HFpEF [12]. Indeed, relative hypophosphorylation of the stiff, N2-B titin-isoform was confirmed in later human experiments, which could be corrected upon *in vitro* administration of PKA or PKG [45], but also in several small (ZSF1-obese rats) and large (old hypertensive dogs) HFpEF animal models [32, 46]. On the other hand, phosphorylation of the PEVK region by PKC increased F_passive_*in vitro* [40], but in the ZSF1-obese rats these specific PEVK sites were not hyperphosphorylated compared with controls [32]. It was recently suggested that hypophosphorylation of the N2-Bus and hyperphosphorylation of the PEVK domain can act complementary to elevate passive tension in failing human hearts [47]. The clinical relevance of this finding for HFpEF needs to be studied in more detail.

**Fig. 4:**
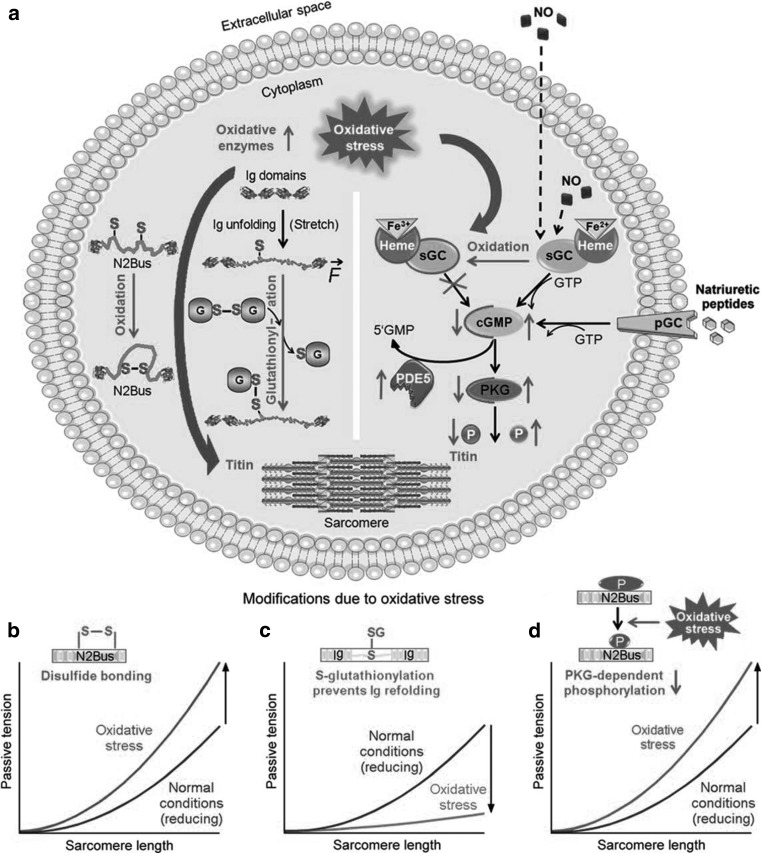
The effects of oxidative stress on titin and cardiomyocyte-based stiffness. **a** Oxidative stress induces post-translational modifications of titin, such as oxidation of cysteines in N2B-unique sequence of titin (N2-Bus) causing disulphide bonding (*far left*), S-glutathionylation of cysteines in unfolded Ig domains inhibiting domain refolding (*left-middle*), and reduced cGMP-dependent protein kinase-G (PKG)–dependent N2-Bus phosphorylation, because of oxidation of the haeme moiety in soluble guanylyl cyclase (sGC) and the ensuing blockade of cGMP production (*right*). Graphs in B to D show oxidative stress–related effects on titin-based passive tension caused by S–S bonding within N2-Bus (**b**), S-glutathionylation of unfolded titin-Ig domains (**c**), or depressed cGMP-PKG pathway activation (**d**). *5′GMP* guanosine-5′-monophosphate, *cGMP* cyclic guanosine monophosphate, *G* glutathione, *GSSG*, glutathione-disulphide, NO, nitric oxide, P, titin phosphorylation, PDE5, phosphodiesterase-5, pGC, particulate guanylyl cyclase, PKG, cGMP-dependent protein kinase-G, and sGC, soluble guanylyl cyclase. (Used with permission from [62])

For HFpEF, especially the relative hypophosphorylation of PKG-dependent titin sites, is an interesting finding that offers potential therapeutic targets (Fig. 4). Not only was titin relatively hypophosphorylated, also PKG activities were shown to be decreased in human HFpEF myocardium, in combination with decreased cyclic guanosine 3’,5’-monophosphate (cGMP) concentration, which activates PKG [48]. In the HFpEF paradigm proposed by Paulus and Tschöpe, decreased cGMP concentration and PKG activity are the final steps in a complex pathway, ultimately leading to increased myocardial F_passive_ and cardiomyocyte hypertrophy [3]. On top of this pathophysiological cascade are metabolic comorbidities such as obesity, diabetes mellitus and arterial hypertension that induce a chronic, inflammatory state, also affecting the coronary microvascular endothelium and leading to oxidative stress [3]. Inflammation and oxidative stress also reduce nitic oxide (NO) bioavailability with subsequently less stimulation of soluble guanylate cyclase (sGC), which catalyses the conversion of guanosine 5’-triphosphate (GTP) to cGMP [49]. Also, direct oxidation of sGC leads to a dysfunctional, haeme-free isoform which is unresponsive to NO [50]. The finding that LV dysfunction and increased myocardial stiffness in diabetic mice is attenuated by the inhibition of dipeptidyl peptidase 4 also supports this paradigm, since these effects are potentially mediated by the stimulation of the cGMP-PKG pathway and the phosphorylation status of titin [51]. This cascade is an important potential target for future HFpEF therapeutic strategies, which will be discussed in detail in another review in this series.

Besides indirect effects via decreased NO-sGC-cGMP-PKG signalling, oxidative stress can also have direct effects on titin-based stiffness (Fig. 4). The N2-Bus, containing 6 cysteines, has a potential to undergo disulphide bonding under conditions of oxidative stress. Indeed, in atomic force experiments it was demonstrated that in the absence of reducing agents, up to three titin-stabilising disulphide bonds could be formed in N2-Bus, leading to a shorter titin length and a secondary increase in F_passive_ [52].

Another part of the I-band contains segments that are rich in immunoglobulin-like (Ig) domains, which make up the majority of elastic titin. Usually parts of these Ig domains are folded into crypts and they can become unfolded and expose cryptic cysteines to disulphide bonding or S-glutathionylation during stretch [31]. A recent study demonstrated that stretching with subsequent S-glutathionylation led to persistent unfolded states, which decreases the mechanical stability of the parent Ig domain as well as its ability to fold and as final result a more extensible state of titin [53]. Especially when stretched cardiomyocytes were incubated with oxidised gluthathione, F_passive_ decreased, whereas incubation with reduced glutathione increased F_passive_, suggesting that also the redox state plays a modifying role in titin-based stiffness that needs to be studied in more detail (Fig. 4).

## Cardiomyocyte and ECM cross-talk

Cardiomyocytes and myocardial ECM are not two completely independent compartments and a close interaction can be expected. Both have been shown to contribute to myocardial stiffness [28] and to be associated with diastolic dysfunction [12, 16, 28]. Interestingly the combination of CVF- and cardiomyocyte-dependent stiffness improved the association of both individual parameters with diastolic dysfunction [12]. Myocardial cells and the ECM can interact at multiple levels. For instance, it has been recently shown that necrotic cardiomyocytes release damage associated molecular patterns (DAMPS) which induce fibroblast activation *in vitro* and myocardial inflammation and fibrosis *in vivo* [54]. Since cardiomyocyte necrosis is more specific to HFrEF [3], future studies are needed to address a potential role for DAMPS in HFpEF. As hypothesised, the pathophysiology of HFpEF starts in the coronary microvasculature, where inflammation and oxidative stress trigger a cascade that affects both the ECM and cardiomyocytes [3]. The importance of the endothelium in HFpEF pathophysiology is also stressed by the finding of coronary microvasculary rarefaction in HFpEF [13].

This interplay between endothelium, ECM and cardiomyocytes has several implications. Firstly, therapeutic options that target either the ECM (e.g. spironolactone) or the cardiomyocytes (e.g. sildenafil), cannot be expected to ‘cure’ HFpEF. Secondly, changes in cardiomyocyte function due to inflammation or oxidative stress may trigger ECM changes and, vice versa, ECM changes caused by chemical or oxidative endothelial-ECM signalling or by mechanical stress can be expected to disturb normal cardiomyocyte functioning [55]. Thirdly, the relative importance of the ECM and the cardiomyocytes to HFpEF pathophysiology is expected to vary during the course of the disease. One could hypothesise that in the early phase of the disease, cardiomyocytes are more determinant of HFpEF pathophysiology and that ECM changes are more relevant in later stages. Indeed, oxidative stress has many very rapid effects on cardiomyocyte function due to the nature of reactive oxygen species [56]. In contrast, with the transition of fibroblasts into myofibroblasts that secrete collagen, the formation of more insoluble and stiffer collagen fibres may take longer before it has a significant effect. For instance ZSF1-obese rats developed an HFpEF phenotype with diastolic dysfunction and elevated filling pressures and an increased myocardial F_passive_ without any change in ECM turnover at 20 weeks of age [32]. On the other hand, in more advanced stages of HFpEF, the ECM seems to be more prominent and capillary rarefaction can be observed [13, 28].

This hypothesis has important therapeutic implications. If cardiomyoctes predominate the initial phases of HFpEF, it is probably useful to improve cGMP-PKG signalling to reduce cardiomyocyte F_passive_ [57]. However, at more advanced stages of HFpEF, correcting cGMP-PKG signalling might have less effects and drugs targeting myocardial fibrosis (e.g. mineralocorticoid receptor antagonists) or more specific processes such as collagen cross-linking (e.g. anti-lysyl oxidase) could prove more effective. This is illustrated by the results from the RELAX trial [58]. In the RELAX trial, chronic treatment with sildenafil was used with the rationale that this would inhibit cGMP breakdown and increase its concentrations, leading to higher levels of PKG. However, sildenafil appeared to have no beneficial effects in this advanced HFpEF population. Therefore, future trials should test different therapeutic strategies based on different phases of HFpEF. Possibly, circulating ECM biomarkers and/or imaging techniques such as T1 mapping with CMR will help in identifying which patients might benefit most from each therapy.

## Conclusions

HFpEF is a complex disease of which the pathophysiology is gradually becoming unravelled. Both structural and functional alterations in cardiomyocytes and the ECM have been reported, leading to increased myocardial F_passive_. However, further mechanistic studies are necessary to elucidate the relative contribution and interplay between the two mechanisms, which may depend on the aetiology and comorbidities as well as on the stage of the disease.

On the other hand, it is essential to develop noninvasive biomarkers for the early identification of the alterations in these two components. For instance, noninvasive techniques such as CMR with T1 mapping and circulating ECM biomarkers are promising, although future studies are needed to assess their potential to truly diagnose, stage or predict outcome and response to therapeutic strategies in HFpEF.

### Funding

This work was supported by a grant from the European Commission (FP7-Health-2010; MEDIA-261409).
